# Arteriovenous Malformation of the Jejunum, Causing Massive Gastrointestinal Bleeding, Treated With Intraoperative Enteroscopy Guidance: A Case Report

**DOI:** 10.7759/cureus.39940

**Published:** 2023-06-04

**Authors:** Sajan Shrestha, Susan Pradhan, Ajay KC, Sujan Shrestha, Prasan Kansakar

**Affiliations:** 1 Gastrointestinal and General Surgery/General Surgery, Pokhara Academy of Health Sciences, Pokhara, NPL; 2 Colorectal Surgery, Clinic NEO, Kathmandu, NPL; 3 Gastrointestinal and General Surgery, Tribhuvan University Teaching Hospital/Institute of Medicine, Kathmandu, NPL; 4 Gastrointestinal and General Surgery, Pokhara Academy of Health Sciences, Pokhara, NPL

**Keywords:** obscure gi bleeding, middle gastrointestinal bleed, intraoperative enteroscopy, hematochezia, bowel disorder, jejunal arteriovenous malformation, operative management, acute gastrointestinal bleed

## Abstract

Arteriovenous malformations of the small intestine are an important differential in cases of occult gastrointestinal bleeding. Localization of the source of gastrointestinal bleeding can be a difficult task, especially in resource-limited settings where balloon-assisted enteroscopy or video capsule endoscopy are unavailable. We herein report the use of intraoperative enteroscopy to help localize and resect a short bowel segment containing a bleeding arteriovenous malformation of the jejunum in a 50-year-old man who presented with hematochezia and pallor leading to hemorrhagic shock. Esophagogastroduodenoscopy and colonoscopy showed no abnormalities, but a contrast-enhanced computed tomography scan of the abdomen revealed a contrast blush in the proximal jejunum. Angiography with coil embolization failed to control his symptoms, and he underwent exploratory laparotomy with intraoperative enteroscopy to try and localize the bleeding, followed by resection of the diseased segment and anastomosis of the small bowel, which led to the successful resolution of the patient’s issues.

## Introduction

Bleeding from the small intestine is relatively rare, accounting for ~five to 10% of all patients presenting with gastrointestinal (GI) bleeding [1]. Vascular lesions, including angiodysplasia, vascular ectasia, and arteriovenous malformations (AVMs), account for approximately 40% of cases of obscure GI bleeding [2]. The true prevalence of AVM could not be found through a search of available databases in Google Scholar and PubMed. A case series by Meyer et al. found 10% of bowel AVMs were located in the jejunum [3]. Small bowel AVMs can be very difficult to diagnose because of their location, even if they are actively bleeding. Bleeding may be either occult or severe, and AVMs have the potential to cause acute, life-threatening hemorrhage [4]. Techniques such as balloon-assisted enteroscopy, capsule endoscopy, and spiral endoscopy greatly facilitate the diagnostic process [5].

Endoscopic therapy and interventional radiology (IR) are the main treatment modalities used to manage these bleeding small bowel vascular lesions [5,6]. Although the diagnostic yield (detection rate) of intraoperative enteroscopy ranges from 55% to 88%, it can be a very useful tool in difficult cases where endoscopy and IR fail to localize and control the bleeding [7]. We present here a rare case of jejunal AVMs diagnosed and managed with the help of intraoperative enteroscopy, resection, and anastomosis of the pathological segment.

## Case presentation

**Figure 1:**
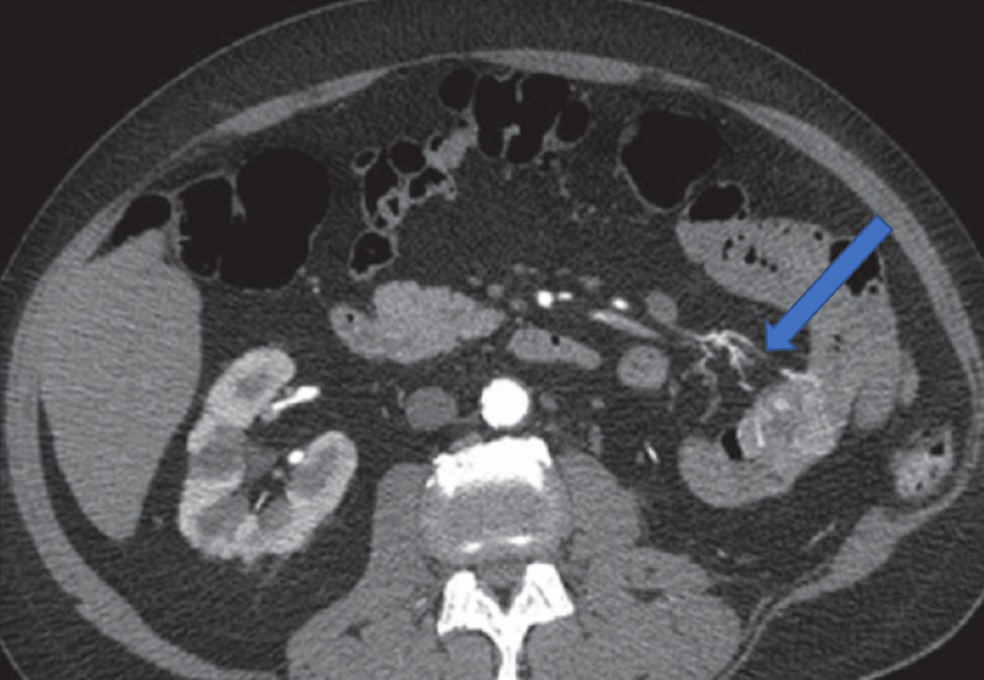
Contrast blush noted in proximal jejunum on CECT abdomen/pelvis

A 50-year-old man presented to our hospital emergency unit with massive hematochezia and pallor. He had a blood pressure of 90/60 mm Hg and a pulse of 112 beats per minute. His hemoglobin (Hb) was five g/dL at presentation. He had gone to another secondary health care center for passage of blood per rectum and generalized weakness and was investigated for a severe GI bleed. His EGD and colonoscopy were normal. Contrast-enhanced computed tomography (CECT) of the abdomen and pelvis was done, which showed contrast blush in the proximal jejunum (Figure 1). He was referred to our institution for interventional radiology consultation, whereupon he underwent conventional angiography with coil embolization in an attempt to control the bleeding. The patient continued to have massive hematochezia along with deterioration of clinical status, with a blood pressure of 90/60 mm Hg, a heart rate of 100 beats per minute, and a fall of Hb to seven g/dL.

**Figure 2:**
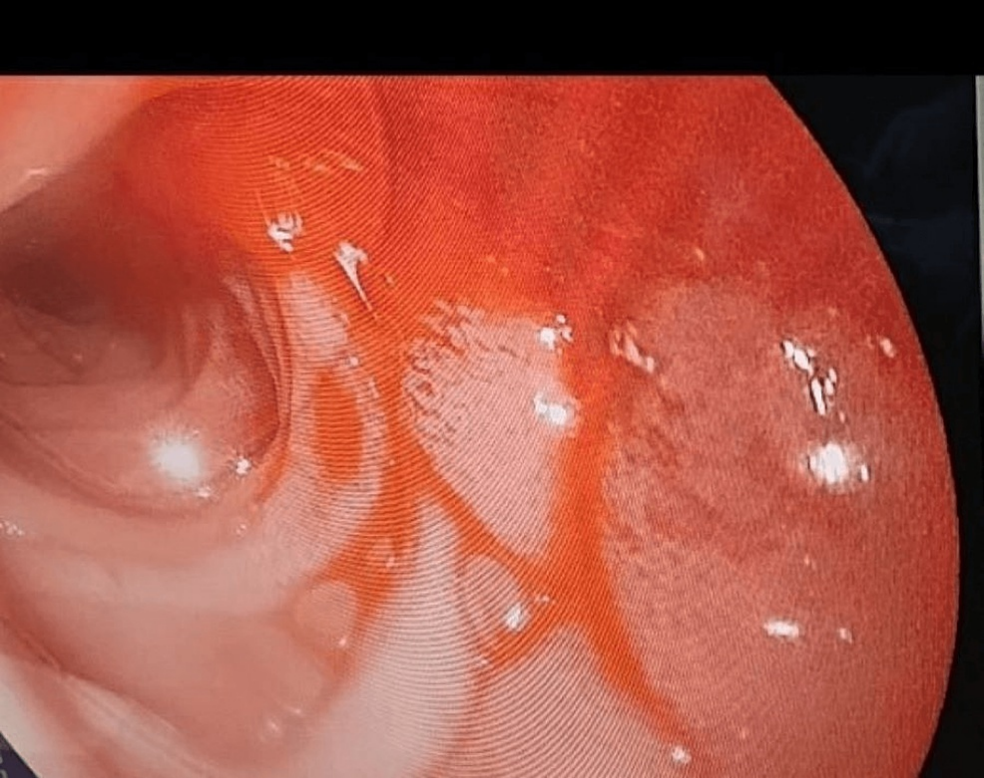
Intraoperative enteroscopy showing pooling of blood in the proximal jejunum

**Figure 3:**
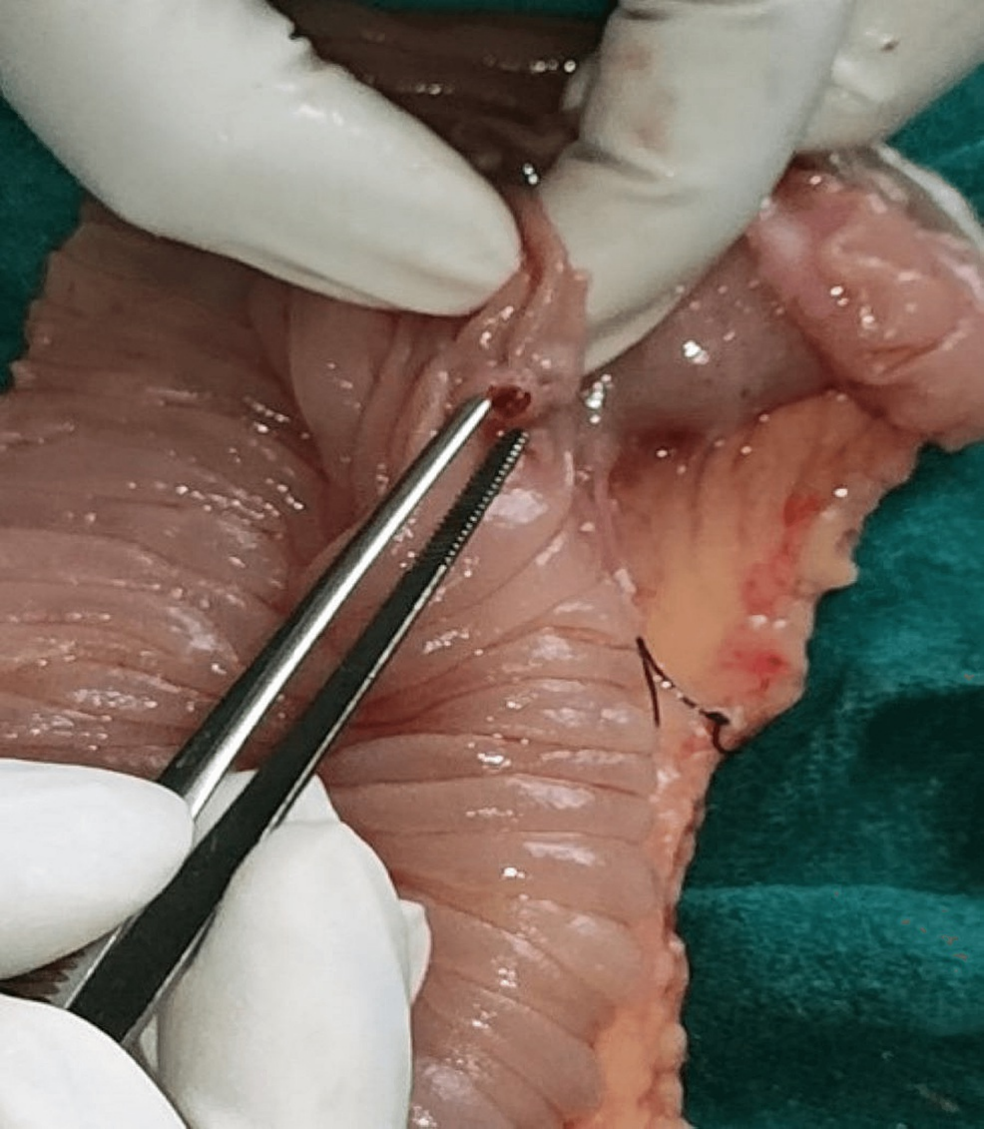
Cut section shows a small vascular lesion in the specimen

**Figure 4:**
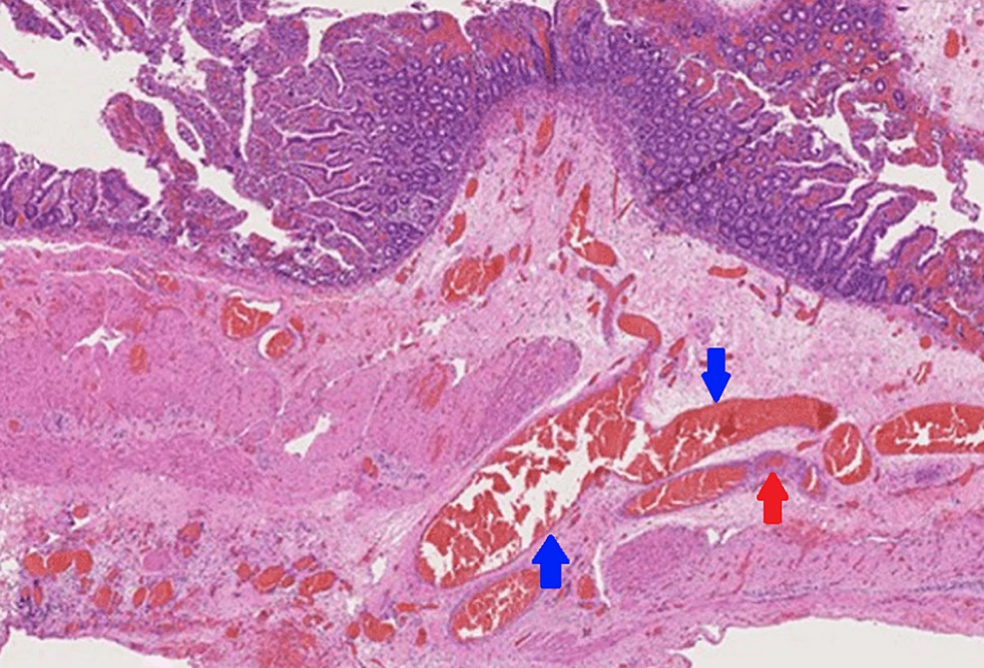
Histopathology of a segment of the jejunum showing arterio-venous malformation Blue arrow: vein. Red arrow: artery.

As per standard protocol, an emergency exploratory laparotomy was indicated and performed within 72 hours of the patient’s presenting to the hospital. Enterotomy was performed approximately 50 cm distal to the duodenojejunal junction (DJ) (site chosen based on possible sites of AVMs detailed in the CECT abdomen). Intraoperative enteroscopy was done distally and proximally to the enterotomy site using a Pentax upper GI scope. No gross lesion was detected, but continuous pooling of blood in the jejunum, approximately 30 cm distal to the DJ junction, was noticed (Figure 2). After a thorough evaluation of the remainder of the jejunum and proximal ileum, resection and anastomosis of approximately 30 cm of the jejunum (10 cm proximal to the pooled blood and five cm distal to the enterotomy site) were done. A small vascular mucosal lesion was noted approximately 10 cm from the proximal end of the resected segment (Figure 3), which was found to be an AVM on histopathological examination (Figure 4). The patient was admitted to the Intensive Care Unit for supportive management, and two units of whole blood were transfused. The patient was discharged on the seventh postoperative day without any complications.

## Discussion

Bleeding from the small intestine is an important differential in cases of obscure GI bleeding [5]. Lesions causing small bowel bleeding may be of vascular origin, like angiodysplasia, AVMs, or due to inflammatory bowel disease, neoplastic growths, or drug-use-related [5]. AVM represents an abnormal connection of arteries and veins and is a rare cause of GI bleeding [8]. Meyer et al. reported that 10.5% of the patients with intestinal AVMs had lesions located in the jejunum among 218 patients [3]. The etiopathogenesis of intestinal AVMs is not fully understood, but increased bowel wall tension is thought to play a role in the acquired variants [4]. Moore classified intestinal AVMs into three categories. Type 1 AVMs are considered an acquired disease, occur mainly in elderly patients, and frequently appear in the right colon. Type 2 AVMs are thought to be congenital and occur in younger patients, typically in the small bowel. Type 3 AVMs are seen in patients with hereditary hemorrhagic telangiectasia [9]. AVMs appear to occur equally in men and women, with no predilection for race [10]. The most common presentation is occult or severe GI bleeding [5]. Mortality risk from severe lower GI bleeding ranges from 2% to 4% [11].

Wireless capsule endoscopy (CE) has become the procedure of choice for the evaluation of obscure GI bleeding, with a diagnostic yield of 49%-63% [12]. Double-balloon enteroscopy can be used to treat lesions identified by CE or as a second-line test when CE is negative [13]. Nonselective or selective angiography is also an important diagnostic tool for detecting bleeding from AVMs of the small intestine [5,10]. Coil embolization of the malformation can be done safely, but the risk of ischemia to the pertaining bowel segment is always there [14]. Surgery is the last resort for severe small bowel bleeding with failed endoscopic and radiological interventions [5]. If the patient needs surgery for treatment of small intestine AVMs, intraoperative localization of the lesions can be difficult [9]. Various case reports and studies exist, showcasing multiple options that can be used to localize these lesions. Defreyne et al. reported the intraoperative use of a methylene blue dye injection in which a microcatheter was utilized to inject methylene blue into the arterial feeding vessel to help localize the lesion [15]. Evans et al. reported the effectiveness of measuring intraoperative mesenteric venous pressure and PO2 in the dilated veins draining the lesions, which tended to be elevated compared to adjacent mesenteric veins, thus aiding in the localization and resection of bowel segments [16]. Ono et al. reported on the use of an intraoperative Indocyanine Green (ICG) dye injection for the localization of AVMs, using intraoperative angiography to inject diluted ICG into the superior mesenteric artery and visualization through ICG fluorescence imaging [17].

In this case, our patient underwent surgical exploration after a failed embolization of the small bowel AVM, with continued bleeding causing hemorrhagic shock. Intraoperative enteroscopy was utilized to narrow down the potential site of bleeding, which enabled a relatively short segment resection, preserving residual bowel length. The patient was hemodynamically unstable; therefore, double balloon enteroscopy and capsule endoscopy were not performed.

## Conclusions

AVM of the small bowel is a rare cause of GI bleeding. Capsule endoscopy, double balloon enteroscopy, and angiography are valuable diagnostic tools. Surgery is the last resort for severe GI bleeding, and intraoperative enteroscopy can be successfully used for localizing the site of small bowel bleeding. Innovative utilization of modern methods of localization is certain to improve our chances of understanding and correctly diagnosing AVMs of the small bowel.
